# The role of a two-assay serological testing strategy for anti-HCV screening in low-prevalence populations

**DOI:** 10.1038/s41598-021-88138-2

**Published:** 2021-04-22

**Authors:** Yanfang Huang, Huifen Pan, Qin Gao, Panpan Lv, Xiaoqin Xu, Zhen Zhao

**Affiliations:** 1 Clinical Laboratory, Minhang Hospital, Fudan University, Shanghai, China; 2 Clinical Laboratory, Shanghai First Maternity and Infant Health Hospital, Shanghai, China

**Keywords:** Microbiology, Gastroenterology

## Abstract

HCV screening depends mainly on a one-assay anti-HCV testing strategy that is subject to an increased false-positive rate in low-prevalence populations. In this study, a two-assay anti-HCV testing strategy was applied to screen HCV infection in two groups, labelled group one (76,442 people) and group two (18,415 people), using Elecsys electrochemiluminescence (ECL) and an Architect chemiluminescent microparticle immunoassay (CMIA), respectively. Each anti-HCV-reactive serum was retested with the other assay. A recombinant immunoblot assay (RIBA) and HCV RNA testing were performed to confirm anti**-**HCV positivity or active HCV infection. In group one, 516 specimens were reactive in the ECL screening, of which CMIA retesting showed that 363 (70.3%) were anti-HCV reactive (327 positive, 30 indeterminate, 6 negative by RIBA; 191 HCV RNA positive), but 153 (29.7%) were not anti-HCV reactive (4 positive, 29 indeterminate, 120 negative by RIBA; none HCV RNA positive). The two-assay strategy significantly improved the positive predictive value (PPV, 64.1% & 90.1%, *P* < 0.05). In group two, 87 serum specimens were reactive according to CMIA screening. ECL showed that 56 (70.3%) were anti-HCV reactive (47 positive, 8 indeterminate, 1 negative by RIBA; 29 HCV RNA positive) and 31 (29.7%) were anti-HCV non-reactive (25 negative, 5 indeterminate, 1 positive by RIBA; none HCV RNA positive). Again, the PPV was significantly increased (55.2% & 83.9%, *P* < 0.05). Compared with a one**-**assay testing strategy, the two**-**assay testing strategy may significantly reduce false positives in anti**-**HCV testing and identify inactive HCV infection in low-seroprevalence populations.

## Introduction

Hepatitis C virus (HCV) infection is one of the leading causes of liver cirrhosis, hepatocellular carcinoma, and mortality worldwide^1–3^. Approximately 71 million people around the world have chronic HCV infection, with an annual increase of 1.75 million new infections^4^. However, only 20% and < 1% of patients were diagnosed and treated in high-income settings and in low- and middle-income countries (LMICs), respectively^5^. Currently, the prevalence of hepatitis C is estimated at 2.8% globally, varying widely in different geographical regions and populations^6^. Among the nations of the world, Egypt is estimated to have the highest HCV prevalence, at 11.9%^7^, whereas Iran has the lowest prevalence, at 0.30%^8^. Most previous studies have focused on screening strategies for populations at high risk for HCV, such as people who inject drugs, men who have sex with men^9–11^, but few studies have paid attention to screening strategies for the general populations.

The World Health Organization (WHO) has developed evidence-based guidelines that focus on who to test and how to test for chronic hepatitis C infection^12^. The current diagnostic algorithm prioritizes the detection of HCV antibodies as evidence of past or current HCV infection through rapid diagnostic tests (RDTs) or laboratory-based serum samples such as enzyme immunoassays (EIAs), electrochemiluminescence immunoassays (ECLs), and chemiluminescence immunoassays (CLIAs). Anti-HCV-reactive serum requires further supplemental testing for HCV RNA or HCV core antigen to confirm whether there is an HCV infection with viraemia.

Several previous studies have attempted to establish the optimal S/CO ratio in various laboratory-based serological assays to minimize the need for supplemental tests and avoid the possibility of reporting false-positive results as much as possible^13,14^. However, the conclusions were not always consistent^14,15^. Only large-scale screening of the general population could substantially accelerate the rate of HCV elimination. A recent study demonstrated that the correlation coefficients of anti-HCV low S/CO ratios were poor between the Architect, Elecsys, and Vitros assays, leading to the inference that combining two serological assays may be useful to eliminate false-positive results^16^. Therefore, our study aims to assess the diagnostic performance of two-assay serological testing strategies in low-prevalence populations.

## Results

### One-assay serological testing strategy using ECL screening

**Figure 1:**
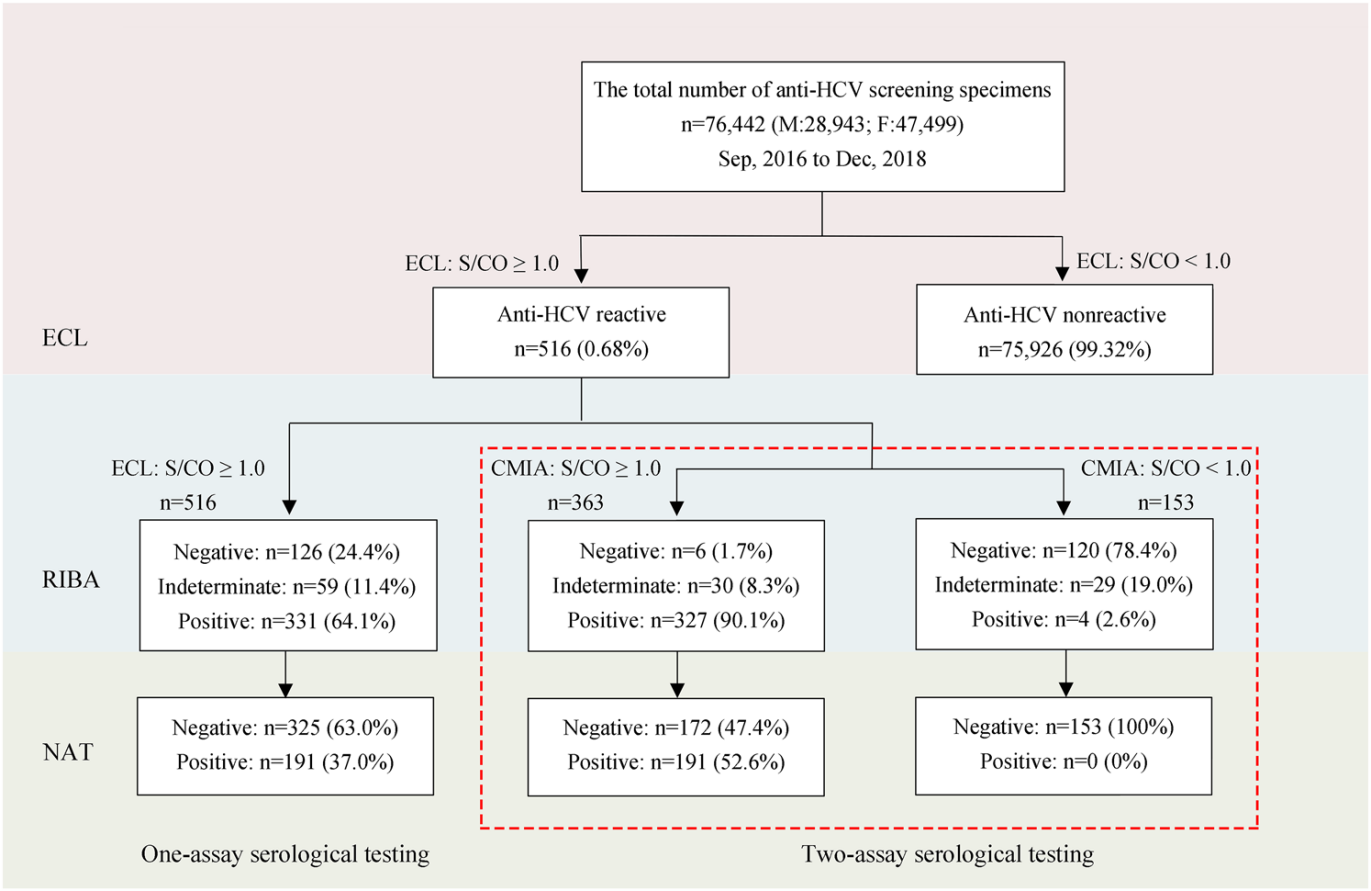
Schematic illustration of the HCV testing sequence for one- or two-assay serological testing strategies (ECL screening and CMIA retest).

Among the 76,442 people in group one, 518 showed anti-HCV reactivity in the ECL assay, of which 2 cases failed to show reactivity in a subsequent repeated test with the same assay (the S/CO values were 1.01 and 1.05, respectively, in the first Elecsys Screening testing, and the RIBA and HCV RNA results were also negative). Therefore, a total of 516 cases were included in this study. Among these sera, RIBA showed that 331 were anti-HCV positive, 59 were indeterminate, and 126 were negative. NAT confirmed that 191 (37.0%) cases were HCV RNA positive (Fig. 1). According to the manufacturer's instructions, samples with S/CO ≥ 1.0 were considered positive for anti-HCV, with a PPV of 64.1% (331/516).

### Two-assay serological testing strategy using ECL screening and CMIA retesting

The 516 specimens that were anti-HCV reactive on ECL were retested using the second serological assay (CMIA), which showed that 363 (70.3%) were anti-HCV reactive, while the other 153 (29.7%) specimens were non-reactive. Among 363 specimens that were anti-HCV reactive on both serological tests, RIBA showed that 327 were anti-HCV positive, 30 were indeterminate, and 6 were negative, for a PPV of 90.1% (327/363), significantly higher than the PPV of the one-assay serological test (Fig. 1, χ^2^ = 94.81, *P* = 0.001). Moreover, 191 cases were HCV RNA positive in the above 363 specimens, which was significantly different from the results of the one-assay serological test (Fig. 1, χ^2^ = 21.11, *P* = 0.001).

Among the 153 sera with inconsistent results, 4 were anti-HCV positive, 29 were indeterminate, and 120 were negative by RIBA (Fig. 1), showing a significant difference compared with the one-assay serological strategy (Fig. 1, χ^2^ = 187.93, *P* < 0.001). Moreover, these 153 specimens were all HCV RNA negative.

### The optimal algorithm for anti-HCV screening with ECL and CMIA

**Figure 2:**
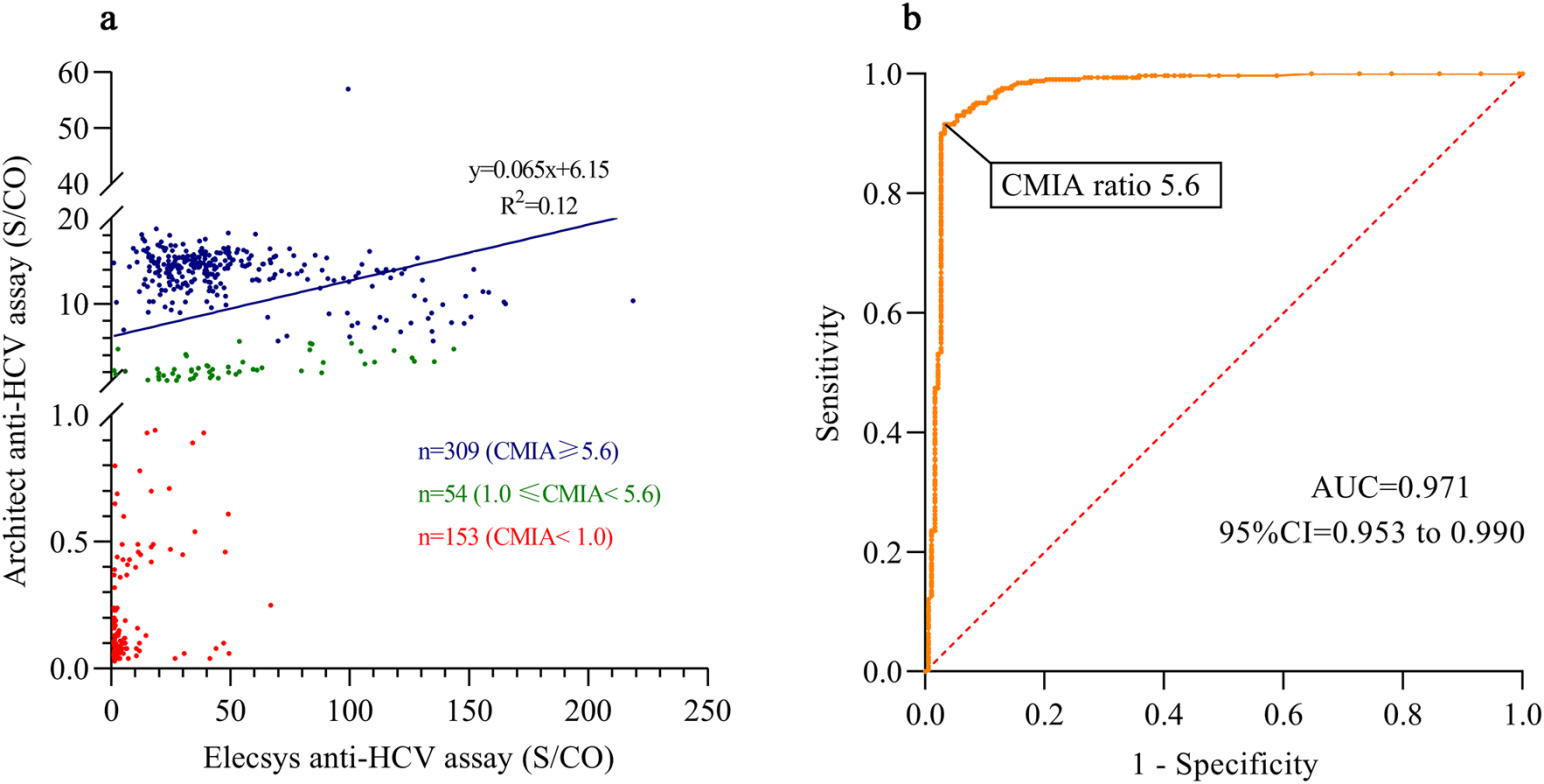
(**a**) Scatter plots of the anti-HCV S/CO ratios of the Elecsys and Architect assays.

Figure 2a shows that the correlation coefficient of the Elecsys and Architect S/CO ratios was low (R^2^ = 0.12). The optimal S/CO ratio of CMIA for anti-HCV positivity was 5.6 according to ROC curve analysis (Fig. 2b). The anti-HCV-reactive specimens were further categorized into three groups according to the results of the two serological assays: ECL ≥ 1.0 and CMIA < 1.0, ECL ≥ 1.0 and 1.0 ≤ CMIA < 5.6, ECL ≥ 1.0 and CMIA ≥ 5.6 (Table 1). Their PPVs were 2.6%, 44.4% and 98.1%, respectively, and the difference was statistically significant (χ^2^ = 415.51, *P* = 0.001). Their HCV RNA positive rates were 0.0%, 5.6%, and 60.8%, respectively, and the difference was significant (χ^2^ = 188.08, *P* = 0.001).

**Table 1:** Predicting the presence of anti-HCV and HCV-RNA using two serological assays.

Classification	Total	RIBA			*P*	NAT		*P*
		Negative	Indeter-minate	Positive		Negative	Positive	
ECL ≥ 1.0 and CMIA < 1.0	153	120	29	4		153	0	
ECL ≥ 1.0 and 1.0 ≤ CMIA < 5.6	54	6	24	24		51	3	
ECL ≥ 1.0 and CMIA ≥ 5.6	309	0	6	303	0.001	121	188	0.001
Total	516	126	59	331		325	191	

*ECL* electrochemiluminescence, *CMIA* chemiluminescent particle immunoassay, *RIBA* recombinant immunccoblot assay, *NAT* nucleic acid amplification testing.

The correlation coefficients of the Elecsys and Architect S/CO ratios was poor (R^2^ = 0.12). (b) Receiver characteristic curve of the RIBA test at different cutoff levels using CMIA for the anti-HCV test. The diagnostic sensitivity and specificity (95% CIs) were 91.5% (88.1–94.1%) and 96.8.7% (93.2–98.5%), respectively, for an S/CO ratio of 5.6. The area under the curve (95% CI) was 0.971 (0.953–0.990).

### Two-assay serological testing strategy using CMIA screening and ECL retesting

**Figure 3:**
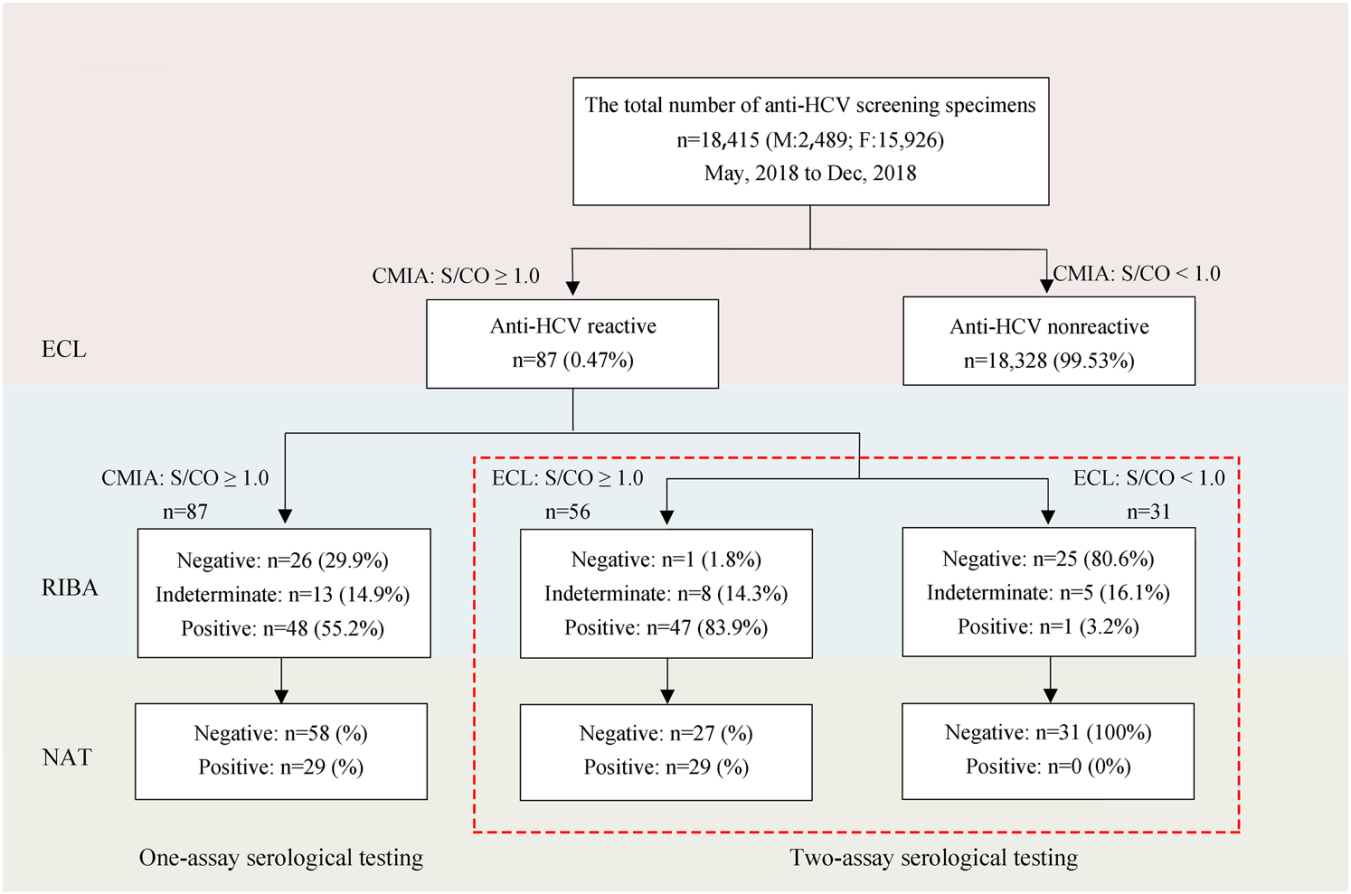
Schematic illustration of the HCV testing sequence for the one- and two-assay serological testing strategies (CMIA screening and ECL retesting).

It was observed that 87 serum specimens in group two (18,415 people) had S/CO ≥ 1.0 using CMIA screening. Of these, RIBA results indicated that 48 were anti-HCV positive (PPV, 55.2%), 13 were indeterminate, and 26 were negative. NAT showed that 29 cases were HCV RNA positive. However, the second serological test (ECL) showed that 56 specimens had S/CO ≥ 1.0 (47 positive, 8 indeterminate, 1 negative by RIBA; 29 HCV RNA positive), and 31 specimens had S/CO < 1.0 (1 positive, 5 indeterminate, 25 negative by RIBA; none HCV RNA positive). The two-assay serological testing strategy improved the PPV from 55.2% to 83.9% (Fig. 3, χ^2^ = 18.50, *P* < 0.01).

## Discussion

Among 76,442 people in group one who were screened with a one-assay testing strategy, 516 (0.68%) were anti-HCV reactive, of which 331 were confirmed to be anti-HCV positive by RIBA, for an HCV prevalence of 0.43% (331/76,442). The prevalence in group two was 0.26% (48/18, 415). Both were lower than the initial screening results. Several similar previous studies in populations with seroprevalence rates of 1.0%, 4.6%, and 20.8% revealed that the corresponding PPVs of the Elecsys anti-HCV II assay were 88.04%, 98.9%, and 99.5%, respectively^17–19^. It is well known that prevalence affects PPV. Some previous studies have shown that the sensitivity of the Elecsys anti-HCV II assay to anti-HCV is relatively high (ranging from 99.3% to 100%)^17,20^. This means that almost all HCV-infected people will be found by serological screening. Nevertheless, even with high specificity (99%), the application of a one-assay test for anti-HCV may lead to a considerable number of false-positive results as well as low PPV, particularly in low-seroprevalence settings or populations^21^.

The causes of false reactivity are diverse, including the interference of many autoantibodies, nonspecific immune responses, antibody cross-reactions to other pathogens, and procedural errors^22,23^. Most manufacturers recommend retesting anti-HCV-reactive sera in duplicate. However, our data suggested that all reactive cases in the first screening test remained anti-HCV reactive in subsequent duplicate testing, except for two borderline results. The consistency rate of the two repeat tests of the Elecsys anti-HCV II assay was 99.6% (516/518). This result suggested that repeated testing using the same assay has no significant ability to discriminate false-positive results.

However, the consistency rate of Roche ECL and Abbott CMIA was only 70.3% (363/516, Fig. 1). This was consistent with Ha J’s report^16^. Surprisingly, using two-assay serological testing, 23.3% (120/516) RIBA-negative cases and 47.1% (153/325) HCV RNA-negative cases were further excluded (Fig. 1). Therefore, our data showed that two-assay serological testing, as an alternative to re-examination in duplicate with the same method recommended by the manufacturer, can significantly reduce false-positive results. Furthermore, compared with a one-assay approach, the two-assay strategy eliminates one test and reduces the cost by 50%, in addition to possibly reducing the number of HCV RNA tests that must be conducted.

Several recent studies have suggested that a high anti-HCV S/CO ratio is accurate at predicting the presence of viraemic HCV infection^24,25^. However, in this study, a high S/CO ratio merely provides insight into the real anti-HCV status and is not able to accurately predict the presence of viraemia, consistent with the report by Ha J et al.^16^. Notably, this would lead to some HCV-infected people being missed if a high anti-HCV S/CO ratio were set as a reference value. However, only 4 specimens were anti-HCV positive by RIBA, and no HCV RNA was positive in the 153 sera that produced discordant results on the two-assay serological tests (ECL S/CO ≥ 1.0 and CMIA < 1.0, Fig. 1).

Moreover, we analysed 87 anti-HCV-reactive sera in CMIA screening at Shanghai First Maternity and Infant Health Hospital. We observed a PPV of 55.2% by RIBA. However, the PPV rose to 83.9% with two-assay serological tests (Fig. 2). Again, this suggests that the two-assay testing strategy could improve the PPV of anti-HCV screening, which would increase the accuracy of epidemiological investigations of HCV infection.

Although the positive predictive value of RIBA is close to 100%, avoiding a high false-positive rate, it is more complicated and expensive than other assays and prone to a higher rate of indeterminate results^26^. NAT technology is also costly, requiring skilled staff and specialized laboratory equipment. Unfortunately, only 60% of anti-HCV-reactive individuals return for HCV RNA testing^27^. A survey of current HCV testing practices in 23 LMICs showed that the majority of these programs provide anti-HCV assays. However, HCV RNA testing is available in only 5% to 30% of countries. HCV core antigen testing is currently not reported in any of these LMICs^28^. The main reasons are lack of public awareness, a low level of knowledge among health professionals, limitations of diagnostic sites, deficiency of funding for HCV testing, and lack of follow-up after the diagnosis of viraemic HCV infection^28^. HCV RNA testing was mainly performed in highly resourced settings, but the majority of HCV-infected individuals were in LMICs. Therefore, a two-assay serological testing strategy may be an excellent alternative strategy to reduce the false-positive rate of anti-HCV testing and rule out inactive HCV infection among anti-HCV-reactive individuals in low-seroprevalence populations. These goals are also increasingly important for accurately assessing the global epidemiology and burden of HCV infection.

In 2016, the WHO set ambitious goals to eliminate hepatitis as a major public health threat. Most countries committed to achieving the bold targets of 90% diagnosis and 80% of diagnosed patients eligible for treatment by 2030^29^. Recently, a new era of highly effective, well-tolerated oral direct-acting antiviral therapy for chronic HCV infection has emerged^30,31^. However, the overwhelming majority of people infected with HCV remain unaware of their infection, and the COVID-19 pandemic has further impacted the elimination of HCV^32^, which has further increased the potentially massive burden of undiagnosed infection worldwide. Therefore, there is an urgent need to establish an effective algorithm for broad-scale diagnosis of potentially HCV-infected individuals while simultaneously maintaining affordable testing costs for individuals and appropriate performance standards for laboratories, which will be critical for curbing the epidemic of HCV worldwide. In order to achieve the 2030 elimination target, the concept of micro-elimination has been proposed; in this strategy, the wider population would be divided into smaller subgroups through targeted treatment and prevention^33^. Similarly, different screening strategies should be targeted and implemented for certain prevalence populations. With the implementation of the hepatitis C elimination plan in 2030, the overall prevalence of HCV infection in the general population will gradually decline^32^. At that time, the two-assay serological test strategy may play an important role in the substantial reduction of false-positive anti-HCV results among low-prevalence populations.

There were several limitations to this research. Since serological tests cannot detect anti-HCV if it is absent or very low in the early stage of HCV infection, there is inevitably a window in which occasional false-negative results occur. Especially in groups with a high HCV prevalence, such as people who inject drugs and men who have sex with men, the two-assay strategy might cause a high number of false negatives. Under these circumstances, NAT is recommended to detect HCV RNA. In addition, the two-assay strategy is not suitable for immunocompromised individuals because they may not be able to produce enough antibodies even if exposed to a high serum HCV load.

In conclusion, one-assay anti-HCV serological tests play an important role in screening for HCV infection, but they also carry an increased risk of false-positive results in low-seroprevalence populations. Supplementary HCV RNA experiments could effectively detect viraemic HCV infection in anti-HCV-reactive individuals, but this method may not be feasible to implement effectively in low-income settings. Our data suggest that two-assay serological testing rather than repeated assays with the same kit could significantly reduce false-positive results and identify inactive HCV infection in HCV screening of low-seroprevalence populations.

## Methods

### Subjects

Two groups of people were included this study. The first group consisted of 76,442 people who planned to receive blood transfusions, surgery^34^ or pregnancy tests^35^ at Minhang Hospital, Fudan University, from September 2016 to December 2018. Among them, 28,943 (37.9%) were males with a mean age of 52.4 ± 17.0 years, and 47,499 (62.1%) were females with a mean age of 42.4 ± 14.5 years. Serum anti-HCV screening of the first group was completed using electrochemiluminescence (ECL) immunoassay systems within four hours after collection. The second group included 2,489 males (37.2 ± 7.5 years old) and 15,926 females (33.2 ± 7.4 years old) who visited Shanghai First Maternity and Infant Health Hospital for blood transfusions, surgery or pregnancy tests from May 2018 to December 2018. Serum anti-HCV screening for the second group was completed using a chemiluminescent microparticle immunoassay (CMIA) within 4 h of collection. Each anti-HCV-reactive serum sample was divided into 3 aliquots (each 500 μl). The first was stored at 4 ℃ and retested within 24 h with the other method. The other two were stored at − 80 ℃ for NAT and RIBA, which were performed as supplemental tests to confirm anti-HCV positivity or active HCV infection within 7 days and 30 days, respectively. All experimental procedures were conducted according to The Declaration of Helsinki; written informed consent was obtained from participants, and the study was approved by the institutional ethics committee of Minhang Hospital, Fudan University.

### ECL immunoassay for anti-HCV

An ECL immunoassay was applied for anti-HCV testing using the Elecsys anti-HCV II assay on the Cobas 601 analyser (Roche Diagnostics, Mannheim, Germany). The kit was a third-generation test using peptides and recombinant antigens representing the core, NS3, and NS4 to capture the corresponding antibodies. The results were expressed as signal-to-cutoff (S/CO) ratios: S/CO < 1.0 indicated anti-HCV nonreactivity, and S/CO ≥ 1.0 indicated anti-HCV reactivity. All S/CO ≥ 1.0 sera were retested in duplicate according to the manufacturer's instructions. If either of the two results remained S/CO ≥ 1.0, then the subject was considered anti-HCV reactive. The anti-HCV reactive sera were retested using an Architect anti-HCV reagent kit.

### CMIA for anti-HCV

A CMIA was performed to test the serum for anti-HCV using an Architect anti-HCV reagent kit on an Architect i2000 analyser (Abbott Diagnostics, IL, USA). The CMIA kit detected HCV antibodies against the fusion protein HCr43 (HCV core antigen and NS3-c33) and the recombinant protein c100-3 (NS4). If S/CO was ≥ 1.0, the serum was retested in duplicate according to the manuals. If either was S/CO ≥ 1.0, the subject was considered anti-HCV reactive and retested using the Elecsys anti-HCV II assay.

### Recombinant immunoblot assay for anti-HCV

Specimens with reactive anti-HCV results were further tested with RIBAs to confirm anti-HCV positivity using a recombinant immunoblot kit for antibodies against the hepatitis C virus (Beijing Wantai Biopharm, Beijing, China). The nitrocellulose strips contained seven bands for the core, NS3, NS4-1, NS4-2, and NS5 antigens as well as control A and control B. The result was defined as negative, ± , 1 + , or 2 + by comparing the colour of the antigen band with control A. Anti-HCV positivity was defined as the presence of at least two antigens with greater than or equal to 1 + (≥ 1 +) reactivity. An indeterminate result was defined by only one band scoring ≥ 1 + . Anti-HCV negativity was defined by the absence of antigens scoring ≥ 1 + . The tests were performed according to the manufacturer’s instructions by technicians with more than 10 years of experience.

### Active HCV infection

Nucleic acid extraction from serum samples was performed with a QIAamp Viral RNA Mini Kit (Qiagen Inc.) according to the manufacturer’s instructions. HCV RNA amplification and quantitative determination were performed using the HCV RNA Quantitation Kit (Shanghai Kehua Bio-Engineering, Shanghai, China), which has a lower limit of detection of 500 IU/mL and is linear from 10^3^ to 10^7^ IU/ml based on TaqMan real-time PCR on the ABI 7500 real-time PCR system (Applied Biosystems, CA, USA). The following PCR program was run: one cycle of UNG enzyme reaction at 50 °C for 2 min and pre-denaturation at 94 °C for 2 min, followed by 40 cycles of denaturation at 94 °C for 10 s and annealing/extension at 60 °C for 30 s.

### Statistical analysis

Statistical analysis was performed with Stata 13.0 software (StataCorp LP, College Station, Texas, USA). The frequency data were analysed using the chi-square (χ^2^) test. Receiver operating characteristic (ROC) curve analysis was performed to evaluate the predictive accuracy of anti-HCV S/CO compared with the RIBA results. Positive predictive value (PPV) is presented as percentages. A *P*-value < 0.05 was considered significantly different.

## Abbreviations

HCV: Hepatitis C virus
anti**-**HCV: Antibody against HCV
ECL: Electrochemiluminescence
CMIA: Chemiluminescent particle immunoassay
RIBA: Recombinant immunoblot assay
PPV: Positive predictive value
S/CO: Signal**-**to**-**cutoff
LMICs: Low**-** and middle**-**income countries
EIA: Enzyme immunoassay
NAT: Nucleic acid amplification testing
CI: Confidence interval
